# Preliminary Pharmacokinetics and Appetite Stimulant Efficacy of Oral Mirtazapine in Guinea Pigs (*Cavia porcellus*)

**DOI:** 10.3390/ani15152256

**Published:** 2025-07-31

**Authors:** Jessica Ayers, Elizabeth Stietzle, Megan Ellis, Jeffrey Kim, Lon V. Kendall

**Affiliations:** 1 Laboratory Animal Resources Department, Colorado State University, Fort Collins, CO 80523, USA; lon.kendall@colostate.edu; 2 College of Veterinary Medicine, Kansas State University, Manhattan, KS 66502, USA; lizstiz@vet.k-state.edu; 3 Laboratory Animal Program, Purdue University, West Lafayette, IN 47907, USA; ellis68@purdue.edu; 4 Comparative Medicine Research Unit, School of Medicine, University of Louisville, Louisville, KY 40208, USA; jeffrey.kim.1@louisville.edu

**Keywords:** appetite, mirtazapine, guinea pig, anorexia, inappetence

## Abstract

Guinea pigs used in research may experience inappetence or decreased intestinal motility, which can significantly compromise their welfare. This study evaluates the use of mirtazapine on appetite and intestinal motility in guinea pigs. An initial pharmacokinetics and efficacy study was performed using healthy male guinea pigs administered mirtazapine at 1.88, 3.75, or 7.5 mg orally once daily for four days (*n* = 6), in a crossover design where all animals received all doses. Body, feed, and fecal weights were taken daily for 4 days. There were no significant differences in weight gains, feed intake, or fecal output as compared to guinea pigs given saline only (*n* = 3). Blood was collected under anesthesia at 0, 0.5, 1, 2, 8, 12, and 24 h post-administration. Pharmacokinetic analysis completed after the first dose showed peak plasma levels at 30 min, then falling below the limit of detection between 8 h and 12 h at all doses. Based on the pharmacokinetic profile, a follow-up study was performed in another set of healthy male guinea pigs with every 8 h dosing at 1.88 mg orally for 5 days (*n* = 6). There was a significant increase in feed intake during mirtazapine administration as compared to baseline intake, but no significant difference in weight gains. This study shows that mirtazapine can be used as an appetite stimulant in guinea pigs but must be dosed at least every eight hours to be effective.

## Simple Summary

Guinea pigs are cecal fermenters requiring frequent and consistent feed intake to ensure normal gut motility. Transport, age-related disease, diet changes, and other sources of chronic stress can reduce their appetite, leading to gastrointestinal stasis which can be life threatening. Mirtazapine, a tetracyclic antidepressant, is used in dogs and cats to treat nausea and inappetence and has been shown to increase feed intake in cats. It has anecdotally been used as an appetite stimulant in guinea pigs, but a therapeutic dose of mirtazapine has not been established. Our work aims to look at the blood levels and physiologic effects of orally administered mirtazapine in guinea pigs.

## 1. Introduction

Guinea pigs are a widely utilized species, acting as research models for infectious disease studies, age-associated arthritis, immunology, nutrition, and many other important human and animal health conditions [1,2,3,4]. Management of guinea pigs in the laboratory is relatively simple since they are docile animals and are easy to house in social groups. However, guinea pigs are hindgut fermenters and herbivorous and require approximately 15% crude fiber in their diet for proper intestinal motility [5]. Guinea pigs may become anorexic from a variety of reasons including illness, dental disease, stress, change in or inappropriate diet, and change in environment or social structure. If they become anorexic, they can rapidly develop gastrointestinal stasis, fluid and electrolyte imbalances, and enterotoxemia, all of which can be fatal [6,7]. Clinical management of gastrointestinal issues should be intensive and include fluid therapy, analgesics, gastric decompression, nutritional support, imaging, and possible surgical intervention [6,7]. Prokinetic therapies, or medications that improve the function or motility of the gastrointestinal system, have also been used and include compounds such as metoclopramide, cisapride, and ranitidine [8]. It is imperative in both clinical practice and research that new drugs are explored as they become available so that care givers have multiple treatment modalities to address this serious health condition to prevent morbidity and death in this species.

Mirtazapine, a presynaptic α2-adrenergic receptor antagonist, originally used in humans as an antidepressant, was noted to have side effects of weight gain and increased appetite. These effects are thought to occur via blockade of the 5-hydroxytryptamine (HT)_Ib_, 5-HT_2,_ and 5-HT_3_ receptors which inhibit the release of peptides involved in appetite stimulation and central nausea induction [9]. The drug was examined in human cancer patients and found to be a promising treatment for managing cancer-related cachexia and anorexia and improving quality of life [9]. Studies in other animal species have focused on these side effects as beneficial treatments for clinical syndromes.

Multiple studies have looked at mirtazapine in cats, administered orally or transdermally, and found significant increases in food intake for both routes [10,11,12]. A study in rats looking at anti-ulcerogenic effects showed mirtazapine to be protective of stress-induced ulcers via increases in extracellular norepinephrine and serotonin levels, although this study did not look specifically at appetite/feed intake [6]. This drug appears to be widely used in dogs to increase appetite, particularly alongside other therapeutics that cause anorexia, such as chemotherapeutic agents. A pharmacokinetic study in healthy research dogs demonstrated that the metabolic profile was similar to humans, but with a much shorter half-life than that found in cats [13]. Another study in dogs demonstrated accelerated gastric emptying in both normal and rectal distension dogs during a 3 h monitoring period via implanted gastric cannulae, along with accelerated colon transit, but not small intestinal transit [14]. Transdermal mirtazapine effects on idiopathic hyporexia in captive macaque monkey species were explored and found to significantly reduce hyporexia frequency for 6 months past the two-week treatment session, with body weights having significantly increased by 6.5 months post-treatment [7].

More recently, mirtazapine has been looked at in New Zealand White rabbits. The oral formulation was found to cause increase fecal output by 25% but did not have significant effects on feed intake, and weights trended down instead of up [15]. However, there may need to be a longer period of administration since studies in humans and mice did not show significant body weight increases until 4–8 weeks and 21 days on the drug, respectively [15]. Another study with rabbits looking at the new transdermal formulation was found to induce a significant increase in feed intake and fecal output versus capromorelin (another appetite stimulant drug) and saline groups in healthy rabbits, but not in post-operative rabbits [16]. Significant gains in body weight versus the saline group were also noted in healthy rabbits in this study [16]. These promising results with other species led to the hypothesis that mirtazapine may increase appetite or intestinal motility in guinea pigs. To this end, the pharmacokinetics and impact of oral mirtazapine on appetite and feed intake was examined in guinea pigs.

## 2. Materials and Methods

### 2.1. Animals

Adult male Hartley guinea pigs (CRL:HA, 550–650 g,) were purchased (Charles River Laboratories, Wilmington, MA, USA) and housed under standard conditions (12:12 h light: dark cycle, 20–70% relative humidity, 69–75 °F), provided ad libitum water (RO filtered) and feed (Envigo Teklad 2040, Envigo, West Lafayette, IN, USA), and socially housed (Allentown static bin caging, 31.875 × 9.875 × 28.00 inches, Allentown, NJ, USA) until the start of the study, then being singly housed (Thoren #6, 12.125 × 23.375 × 9.00 inches, Hazleton, PA, USA) for the duration of the study for experimental measurements.

### 2.2. Initial PK and Efficacy Study

For the initial pharmacokinetic and efficacy studies, 9 animals were used—6 treatment and 3 control animals. The 3 controls were not medicated but had body weight, fecal weight, and feed intake monitored as a comparison to treatment animals in order to account for normal growth over time since each treated guinea pig experienced all 3 doses in a cross-over design leading to a study timeline of 41–45 days where the young guinea pigs were naturally gaining weight as they grew.

Three sessions utilizing different doses (1.88 mg, 3.75 mg, and 7.5 mg) were performed in a crossover design with a one-week washout between doses for all guinea pigs, and doses within each session were randomized (radomizer.org) amongst the animals. At baseline, the 6 treatment animals had a mean ± SD weight of 615.9 ± 20.52 g and the 3 control animals had a mean ± SD weight of 655.2 ± 6.9 g. Doses were chosen based on work performed in cats recommending 1.8–3.75 mg per cat [12]. Since rodents are known to often have higher dosing requirements than larger species, the 7.5 mg dose was also included. Blood (0.5 mls) was collected under isoflurane (Fluriso, USP, Vet One, MWI, Boise, ID, USA) anesthesia from the cranial vena cava at Day 0 (pre-mirtazapine), then at 30 min, 1 h, 2 h, 8 h, 12 h, and 24 h after the oral administration of the first dose of mirtazapine. Half of the guinea pigs were collected at time points 0, 1 h, 8 h, and 24 h, while the other half were collected at 0, 30 min, 2 h, and 12 h, (total blood volume per animal per session = 2.0 mL, repeated every 16–18 days for two more sessions) in order to stay below the IACUC maximum volume withdrawal of 10% total blood volume every 3–4 weeks for any one animal. Doses were administered by gentle manual restraint and placing the 1 mL slip tip syringe into the side cheek and gently pushing the suspension into the mouth (0.25, 0.5, and 1 mL, respectively). Subsequent mirtazapine doses were administered every 24 h for 4 more days and animal, feed, and fecal weights were recorded daily during dosing and then for an additional 4 days post-dosing, followed by a 7–9 day washout between doses. This process was repeated two more times so that all animals were give all doses.

Animals were also observed in cage with no handling for 5–10 min each day to monitor for any subjective adverse effects such as sedation, hyperactivity, pica, etc. Animal, feed, and fecal weights were averaged and compared across the pre-, peri-, and post-mirtazapine administration periods to determine if there was increased weight gain, feed intake, or higher fecal output during drug treatment compared to non-drug controls.

### 2.3. Second Efficacy Study

Based on the pharmacokinetic profile, a second efficacy study was performed using six additional guinea pigs, mean ± SD weight of 565.21 ± 6.97 g at baseline. The lowest dose was chosen since the initial pharmacokinetic study demonstrated similar kinetics for all three doses and the smaller volume was easier to administer. Baseline animal and feed weights were monitored for 5 days prior to dosing as pre-treatment comparison, and mirtazapine was then administered orally via syringe at 1.88 mg (0.25 mL) every 8 h for 5 days. Animal and feed weights were then monitored an additional 6 days as the post-treatment comparison. Fecal output was not measured during this portion due to no effects seen in the first study. Pre and post administration animal and feed weights were compared to the mirtazapine administration period to determine if there was increased feed intake or weight gain during drug treatment.

### 2.4. Drug Preparation

Mirtazapine tablets (15 mg, Aurobindo Pharma USA, Inc., Dayton, NJ, USA) were compounded in-house by a single investigator by adding one 15 mg tablet to 1 mL 50% sterile dextrose solution plus 1 mL 0.9% NaCl solution and mixing continuously until tablets had dissolved evenly in the fluid (final concentration 7.5 mg/mL), based on the protocol used by Ozawa et al. in rabbits [15]. Preparations were mixed vigorously prior to each dose withdrawal to ensure even distribution of drug and were made up daily with no storage of solution occurring.

### 2.5. Mirtazapine Analysis

Blood samples were centrifuged at 3500× *g* for 15 min, and plasma was harvested and placed in storage at −80 °C until analysis (Pharmacology Shared Resource, Colorado State University, Fort Collins). Mirtazapine was measured using liquid chromatography coupled to tandem mass spectrometry (LC/MS/MS) by the Pharmacology Laboratory of the Drug Discovery & Development Shared Resource for the University of Colorado Cancer Center using their standard protocols described below. Standard, quality control (QC), and unknown samples were prepared using a 50 μL aliquot of blank (standard and QC samples) or unknown plasma in a 1.5 mL polypropylene microcentrifuge tube and adding 5 μL of 100 ng/mL trazodone (internal standard) and 5 μL of mirtazapine standard (5–2500 ng/mL) to standard and QC samples or 5 μL of acetonitrile: water to unknown samples. Proteins were then precipitated by the addition of 50 µL of acetonitrile and vortexing for 10 min. Samples were centrifuged at 13,000 RPM for 10 min and the resulting supernatant collected for analysis.

Positive ion electrospray ionization mass spectra were obtained with a 3200 QTRAP triple quadrupole mass spectrometer (Applied Biosystems, Foster City, CA, USA) with a turbo ion spray source interfaced to a 1200 Series Binary Pump SL liquid chromatography system (Agilent Technologies Inc., Santa Clara, CA, USA) and HTC-PAL autosampler (Leap Technologies, Carrboro, NC, USA). Chromatography was performed on a SunFire C8, 2.5 µm, 4.6 × 50 mm column (Waters Corporation, Milford, MA, USA) with a liquid chromatography gradient employing 10 mM ammonium acetate (mobile phase A) and acetonitrile (mobile phase B). Initial chromatographic conditions were 60:40 (mobile phase A: mobile phase B) with increasing B from 40% to 98% from 0 to 2 min, holding at 98% B from 2 to 3.5 min, and re-establishing initial conditions from 3.5 to 5 min. The flow rate was 1.2 mL/min, injection volume was 30 µL, and the total analysis time was 5 min. The mass spectrometer settings were optimized for multiple reaction monitoring transitions for mirtazapine (*m/z* 266→195) and trazodone (*m/z* 372→176) using optimization protocols within Analyst Software (AB Sciex V. 1.7) with turbo ion spray temperature of 600 °C and ion-spray voltage of 5500 V.

Assay performance for each batch was assessed utilizing at least 10% QC samples dispersed amongst unknown samples at low (1 ng/mL), mid (10 ng/mL) and high (100 ng/mL) ranges of the standard curve (0.5–500 ng/mL). Accuracy of QC samples amongst the batches analyzed for this study gave accuracy and precision (%RSD) of 93.0% ± 4.3% with 15/15 QC samples passing with >85% accuracy. The accuracy is calculated as Abs (observed-actual)/actual × 100.

Zero was used when results were reported as “below LLOQ” for calculations. LLOQ was defined as <0.5 ng/mL.

Noncompartmental analysis was performed on plasma mirtazapine concentrations using commercially available software (Phoenix Winnonlin, Certara, Princeton, NJ, USA).

### 2.6. Statistical Analysis

Based on a 2011 article investigating mirtazapine in cats [12] (Quimby), which reported dosed cats consumed feed an average of 56% over baseline versus placebo consumption of 13%, we used a standard deviation of 20% and a conjectured within GP correlation of 0.5, so that *n* = 6 animals would allow for a power of >95%. 

For the initial efficacy study, body, feed, and fecal weights were averaged for each dosing session and analyzed via a two-way repeated measures ANOVA with a post hoc Tukey adjusted pairwise comparison between drug stages and controls (IBM SPSS Software, V. 10.1.6).

For the follow-up study, body weights and feed weights were averaged and results were analyzed using a two-way repeated measures ANOVA (JMP Software Version 14.0) to compare between drug stages. A *p* value < 0.05 was considered statistically significant.

## 3. Results

### 3.1. Pharmacokinetics

**Figure 1:**
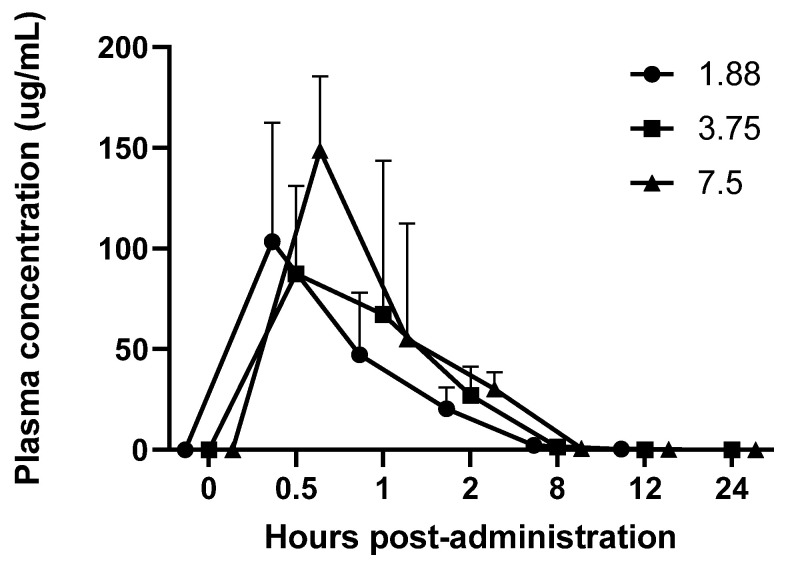
Mean (+ SD) plasma mirtazapine concentrations (µg/mL) after 1.88 mg, 3.75 mg, and 7.5 mg oral dosing in male guinea pigs.

Regardless of dose, mirtazapine blood levels followed similar kinetics across all doses (Table 1). There was a peak in blood levels (average ± SD) at 30 min for all three doses, at 103.3 ± 59.0 ng/mL at the 1.88 mg dose, 87.4 ± 43.7 ng/mL for the 3.75 mg dose, and 148.7 ± 36.8 ng/mL for the 7.5 mg dose, and. Levels fell to 2.2 ± 2.4 ng/mL, 1.4 ± 1.6 ng/mL, and 0.8 ± 0.4 ng/mL, respectively, at the 8 h time point for all three doses and was undetectable at 12 and 24 h post administration (Figure 1).

### 3.2. Initial Efficacy

**Figure 2:**
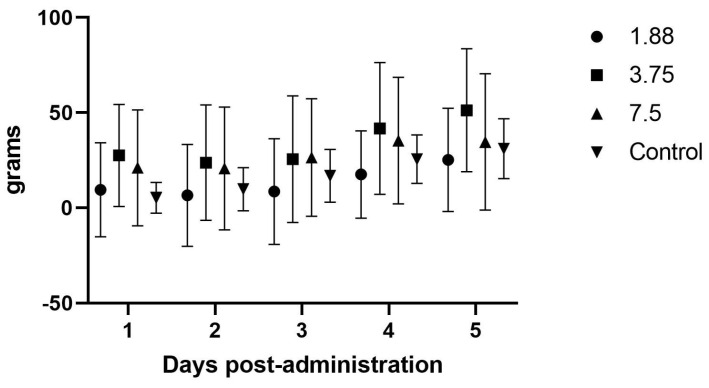
Average (± SD) daily guinea pig weight change (g/day) during once daily oral mirtazapine dosing.

**Figure 3:**
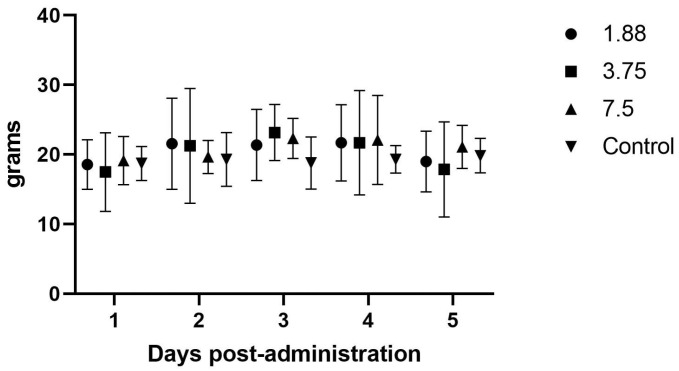
Average (± SD) daily guinea pig fecal weights (g/day) during once daily oral mirtazapine dosing.

**Figure 4:**
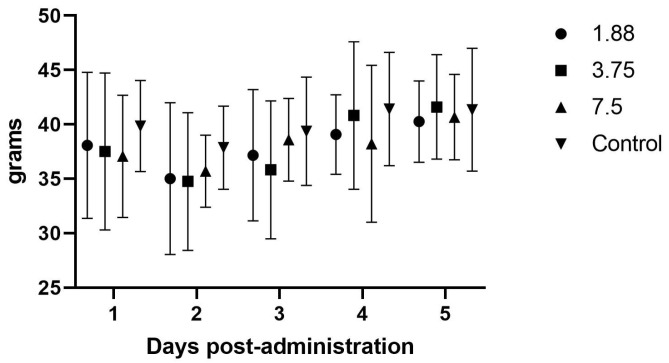
Average (± SD) daily guinea pig feed intake (g/day) during once daily oral mirtazapine dosing.

The initial efficacy study evaluated the effects of increasing dosages on the body weight, feed intake, and fecal output following daily treatment for four days. In this initial study with once daily dosing, there were no significant differences noted in weight gain (*p* = 0.42), fecal output (*p* = 0.49), or feed intake (*p* = 0.67) between the drug doses versus non-drug groups (Figure 2, Figure 3 and Figure 4). There were no abnormalities noted on behavioral observations post-administration in any group.

### 3.3. Second Efficacy Study

**Figure 5:**
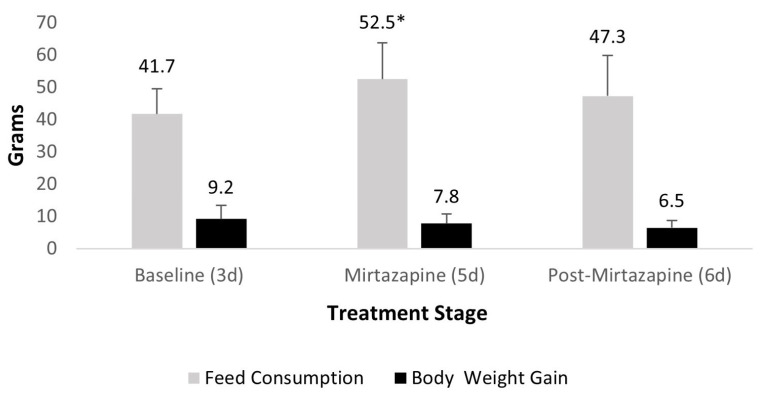
Average (+ SD) daily guinea pig feed consumption and weight gain during q. 8 h oral mirtazapine dosing, * denotes significance compared to baseline.

In the second efficacy study based on the PK profile using the 1.88 mg/kg dosage, there was no significant difference in body weight changes between the three treatment periods (*p* = 0.77). However, compared to baseline feed consumption, mirtazapine treatment resulted in a 21% increase in consumption (*p* = 0.002) (Figure 5).

## 4. Discussion

Guinea pigs become anorexic for a variety of reasons including stress, transport, pain, surgery, medication administration, and other illness. The anorexia can quickly lead to gastrointestinal stasis and dysbiosis that can cause death, so it is essential that multi-modal therapy is instituted to correct the anorexia as rapidly as possible. Prokinetic drugs are often helpful during this phase and the addition of mirtazapine may be of great benefit to the management of this species, both in clinical practice and in research colonies.

### 4.1. Pharmacokinetics

This study found that mirtazapine administered at 1.88 mg, 3.75 mg and 7.5 mg orally had a similar pharmacokinetic profile with a peak plasma concentration at 30 min and undetectable levels at 12 h post-administration. Higher doses appeared to have a slight duration effect, where blood levels were slightly higher at the 8 h time point than the lowest dose, although not significantly.

The PK profile did show some variability, particularly in the half-life of the 3.75 mg dose, which calculated as much longer than the lower and higher doses. We suspect that this variability was due to the oral administration of the drug as well as the drug being formulated in-house with the possibility of variable solution concentrations at time of administration. We made every attempt to evenly mix the drug prior to every dose withdrawal, but there was precipitation of the drug that was noted so it is possible that some animals received a higher dose than intended.

We also gave each animal a standard dose, but this was not based on specific animal weights, so mg/kg dose was variable between individuals. Based on the baseline weight of the treated animals being 615.9 ± 20.52 g, this resulted in a minimum dosing range of 3.0–3.2 mg/kg for the 1.88 mg dosing, 6.0–6.3 mg/kg for the 3.75 mg dosing, and 12.0–12.6 mg/kg for the 7.5 mg dosing, which may have also contributed to PK variability.

### 4.2. Efficacy Studies

There were no effects noted in feed intake, weight gain, or fecal output in the initial once daily dosing efficacy study, which makes sense in light of the PK analysis showing blood levels dropping to zero around the 8 h time point, and is further supported by the significant increase in feed intake when the drug was administered every 8 h. Although significant weight gains were not seen in this study, the most important aspect of guinea pig illness usually is the need to have feed or fiber in the gastrointestinal tract so that it can begin to function normally. The administration of mirtazapine appears to support this by causing the guinea pig to increase its feed intake and we therefore would expect this to be a useful therapy for this species. Some limitation in the methods used were the young age and continued growth of the guinea pigs during the study phases, which may have affected the weight gains and appetites recorded here. We also only monitored feed intake at 24 h intervals, but there may have been more acute affects from the drug immediately after dosing that were not captured. If further studies occur, it would be prudent to include more frequent feed intake measurements, such as every 1–2 h after administration, to look for a dose effect situation where the animal is eating the most when the plasma level is above some threshold (a therapeutic level) and then the intake drops off as the level drops off. Intensive care, such as dosing every 3–4 h in the early stage of management, could encourage greater intake and trigger a more normal appetite even in the face of lower plasma levels when dosing frequency decreases, which could be more feasible for practitioners.

We also used short periods of isoflurane anesthesia for the blood collections in the initial study with each animal having four anesthetic periods within the first 24 h of administration for each phase, which can have negative effects on feed intake and weight gain and could have contributed to a lack of response. We did not use anesthesia in the second efficacy study; however, we were handling and dosing the animals three times daily which could have caused a stress response that affected feed intake similar to anesthesia administration and those differences would be good to explore in future studies.

Another consideration is that the drug doses used in this study were extrapolated from studies in other species, however rodents are known to have much higher dose requirements than other species for many drugs, and higher doses may show an improved PK profile compared to that seen in this study [17]. The drug formulation was also compounded in house and it is possible that variability in drug distribution was present, allowing for variable dosing between animals. It would be prudent to analyze the compounded fluid for drug concentration accuracy in future studies. Since we did not note any adverse side effects of mirtazapine, such as the sedation seen in humans, in this species even at the higher dosage, it may also be warranted to perform PK profiles after administration of higher doses of the drug to explore whether this could prolong the therapeutic effect.

We did not analyze specific tissue distributions of this drug in this study, only serum levels, however other studies have considered the issue that you need drugs to be adequately distributed to target tissues in order for clinical effects to be maximized so it may provide more valuable information if brain tissue is analyzed to see how well this drug crosses the blood–brain barrier and if there are dose effects present there [18]. Additionally, one study looking at pharmacokinetics in rats showed significant sex differences in rate of metabolism and clearance, and although appetite stimulation effects were not evaluated, sex differences should be considered in future efficacy experiments [19].

Studies in clinically ill animals are also needed to see whether the appetite stimulation effect will be present in clinically relevant ways. Since the time of this study, the Food and Drug Administration has approved a commercial transdermal formulation for use in cats (Mirataz, Dechra Veterinary Products, Leawood, KS, USA and it has shown promise in rabbit studies [16]. This formulation could be beneficial in guinea pigs to decrease the stress of handling and dosing, which can exacerbate the anorexia we are trying to treat.

## 5. Conclusions

Mirtazapine, a presynaptic α2-adrenergic receptor antagonist, originally used in humans as an antidepressant, has been used in many other species as an appetite stimulant. This study showed that the drug must be given at least every 8 h in guinea pigs in order to maintain detectable blood levels, and at this dosing level, it does induce a significant increase in feed intake in young healthy male guinea pigs, although significant weight gain was not seen.

## Figures and Tables

**Table 1:** Noncompartmental analysis of mirtazapine pharmacokinetics variable after oral administration at 1.88, 3.75, 7.5 mg in male guinea pigs.

		Dose (mg)		
Parameter	Units	1.88	3.75	7.5
λz	1/h	0.3	0.5	0.6
HL λz	h	1.98	1.31	1.14
T_max_	h	0.5	0.5	0.5
C_max_	μg/mL	103.3	87.4	148.7
AUC_last_	h × μg/mL	171	193	224

λz, elimination rate constant; HL λz, terminal half-life; T_max_, time of maximum concentration; C_max_, maximum concentration; AUC_last_, area under the concentration-time curve from the time of dosing to the last measurable concentration.

## Data Availability

Please contact primary author for data requests.

## Abbreviations

The following abbreviations are used in this manuscript:

LLOQ	Lower Limit of Quantification
HT	Hydroxytryptamine
CRL:HA	Charles River: Hartley
RO	Reverse osmosis
NaCL	Sodium chloride
LC	Liquid chromatography
MS	Mass spectrometry
QC	Quality Control
RPM	Revolutions per minute
ANOVA	Analysis of Variance
SD	Standard deviation
